# Metabolic engineering of *Bacillus amyloliquefaciens* for poly-gamma-glutamic acid (γ-PGA) overproduction

**DOI:** 10.1111/1751-7915.12136

**Published:** 2014-07-01

**Authors:** Jun Feng, Yanyan Gu, Yang Sun, Lifang Han, Chao Yang, Wei Zhang, Mingfeng Cao, Cunjiang Song, Weixia Gao, Shufang Wang

**Affiliations:** 1 Key Laboratory of Molecular Microbiology and Technology for Ministry of Education, Nankai UniversityTianjin, China; 2 State Key Laboratory of Medicinal Chemical Biology, Nankai UniversityTianjin, China; 3 Department of Chemical and Biological Engineering, Iowa State UniversityAmes, IA, USA

## Abstract

We constructed a metabolically engineered glutamate-independent *Bacillus amyloliquefaciens* strain with considerable γ-PGA production. It was carried out by double-deletion of the *cwlO* gene and *epsA-O* cluster, as well as insertion of the *vgb* gene in the bacteria chromosome. The final generated strain NK-PV elicited the highest production of γ-PGA (5.12 g l^−1^), which was 63.2% higher than that of the wild-type NK-1 strain (3.14 g l^−1^). The γ-PGA purity also improved in the NK-PV strain of 80.4% compared with 76.8% for the control. Experiments on bacterial biofilm formation experiment showed that NK-1 and NK-c (Δ*cwlO*) strains can form biofilm; the *epsA-O* deletion NK-7 and NK-PV strains could only form an incomplete biofilm.

## Introduction

Poly-γ-glutamic acid (γ-PGA) is an unusual homopolymer of D/L-glutamate acid polymerized through γ-glutamyl bonds (Ashiuchi and Misono, 2002). γ-PGA is biodegradable, edible, water-soluble and non-toxic toward humans and the environment. Therefore, it has attracted wide interests in a broad range of fields: food, medicine and water treatment (Shih and Van, 2001; Ashiuchi, 2013).

γ-PGA-producing strains are divided into two categories based on their requirement for exogenous glutamic acid: glutamate-dependent strains and glutamate-independent strains (Shih and Van, 2001). Compared with the glutamate-dependent strains, the independent strains are more preferable for the industrial production of γ-PGA with their low cost and simplified fermentation process (Cao *et al*., 2011). However, their lower γ-PGA productivity compared with glutamate-dependent strains limits their industrial application. Construction of a metabolically engineered glutamate-independent strain with high γ-PGA productivity is required.

*Bacillus amyloliquefaciens* NK-1 is a derivative of *B. amyloliquefaciens* LL3 (Cao *et al*., 2011). It is a glutamate-independent strain that produces γ-PGA comprising units of L-glutamic acid and D-glutamic acid. The endogenous plasmid pMC1 and *upp* gene of the NK-1 strain has been deleted, so it is suitable for genetic manipulation such as markerless deletion or insertion of genes.

The approaches employed for the enhancement of γ-PGA production has been mainly focused on two aspects: (i) optimization of the fermentation conditions (Bajaj and Singhal, 2009; Huang *et al*., 2011) and (ii) characterization of the genes involved in γ-PGA production and use of bioengineering methods to improve the γ-PGA yield (Ashiuchi *et al*., 2006; Scoffone *et al*., 2013; Zhang *et al*., 2013). Increasing numbers of studies have focused on the second approach to construct metabolically engineered microorganisms with high γ-PGA productivity. Heterologous expression of the γ-PGA synthetase complex (*pgsBCA*) is another strategy for γ-PGA production improvement, and it has been carried out in coryneform bacteria and *Escherichia coli* (Sung *et al*., 2005; Cao *et al*., 2013).

Peptidoglycan hydrolases belong to the NLPC/P60 family (DL-endopeptidase family II), such as LytE, LytF, CwlS and CwlO (Smith *et al*., 2000; Bisicchia *et al*., 2007; Vollmer *et al*., 2008). These enzymes can not only hydrolyse peptidoglycan, but also degrade γ-PGA. Single deletions of *lytE*, *lytF*, *cwlS* and *cwlO* were studied in glutamate-dependent *Bacillus subtilis* (*natto*), and only the *cwlO* deletion strain showed higher productivity of γ-PGA, and disruptions of other genes had little effect (Yamaguchi *et al*., 2004; Mitsui *et al*., 2011).

Exopolysaccharides (EPS) are synthesized primarily in microbial cells and then secreted into the extracellular environment as biofilms and capsules (Branda *et al*., 2001; 2005; Marvasi *et al*., 2010; Donot *et al*., 2012). Some *Bacillus* strains can produce abundant EPS and are essential for the formation of biofilms (Donot *et al*., 2012). The *epsA-O* cluster has been reported to be associated mainly with EPS production (Branda *et al*., 2004; 2006), which are the main by-products of some γ-PGA-producing strains. Thus, deletion of *epsA-O* can depress EPS production, and metabolic flux could be used to enhance γ-PGA productivity.

*Vitreoscilla* hemoglobin (VHb) is one of the most extensively studied bacterial hemoglobin molecules synthesized by the Gram-negative bacterium *Vitreoscilla* (Wakabayashi *et al*., 1986). It has been confirmed that VHb can interact directly with the terminal respiratory oxidase by delivering oxygen to it, in order to enhance oxidative phosphorylation, and thus the production of adenosine triphosphate (Park *et al*., 2002). It has been demonstrated that VHb expression in heterologous bacterial hosts can enhance cell density and metabolic production, especially under oxygen-limiting conditions (Kang *et al*., 2002; Urgun-Demirtas *et al*., 2003). Heterologous expression of the *vgb* gene (which encodes VHb) has been conducted in various microorganisms as well as plant and mammal cells (Zhang *et al*., 2007). During the fermentation process of γ-PGA, broth viscosity increased and dissolved oxygen in the broth reached 0% after 12–18 h of incubation, which decreased oxygen transfer to cells, inhibited cell growth and γ-PGA production. Expression of the *vgb* gene in a γ-PGA-producing strain will alleviate oxygen limitation at the later stage of fermentation to enhance cell density and γ-PGA production.

In the present study, we aimed to construct a high-productivity γ-PGA-producing strain based on the glutamic acid-independent *B. amyloliquefaciens* NK-1. We double-deleted the *cwlO* gene and *epsA-O* cluster and simultaneously inserted the *vgb* gene in the bacterial chromosome. The generated engineered strain NK-PV showed higher production of γ-PGA and γ-PGA purity compared with the wild-type NK-1 strain.

## Results

### Identification of target gene knockouts or insertion

To construct a metabolically engineered, γ-PGA high-producing strain, double-deletion of the *cwlO* gene and *epsA-O* cluster and insertion of a *vgb* gene were carried out in the *B. amyloliquefaciens* NK-1 strain. A gene marker-less knockout method was used to construct the gene deletion and insertion mutant, which was based on an *upp* cassette and the 5-FU selection (Keller *et al*., 2009).

**Figure 1:**
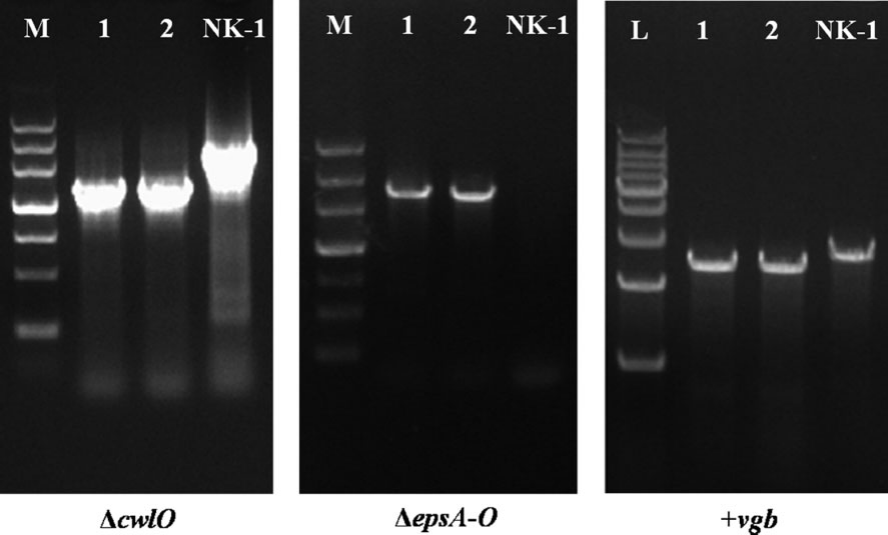
Confirmation of the gene deletion or insertion by PCR. Chromosomal DNA served as the template for amplification. Lane M, DNA marker III; Lane L, DNA ladder (1 kb); Lane 1, 2, gene deletion or insertion strains amplified with relative primers; Lane NK-1, wild-type NK-1 strains amplified with relative primers.

The primers cwlO-SS/XX, AO-SS/XX and Amy-SS/XX were used to verify the gene mutants (Fig. 1). DNA sequencing was used to confirm construction of the engineered strain.

### Confirmation of expression of the *vgb* gene and its effect on cell growth

**Figure 2:**
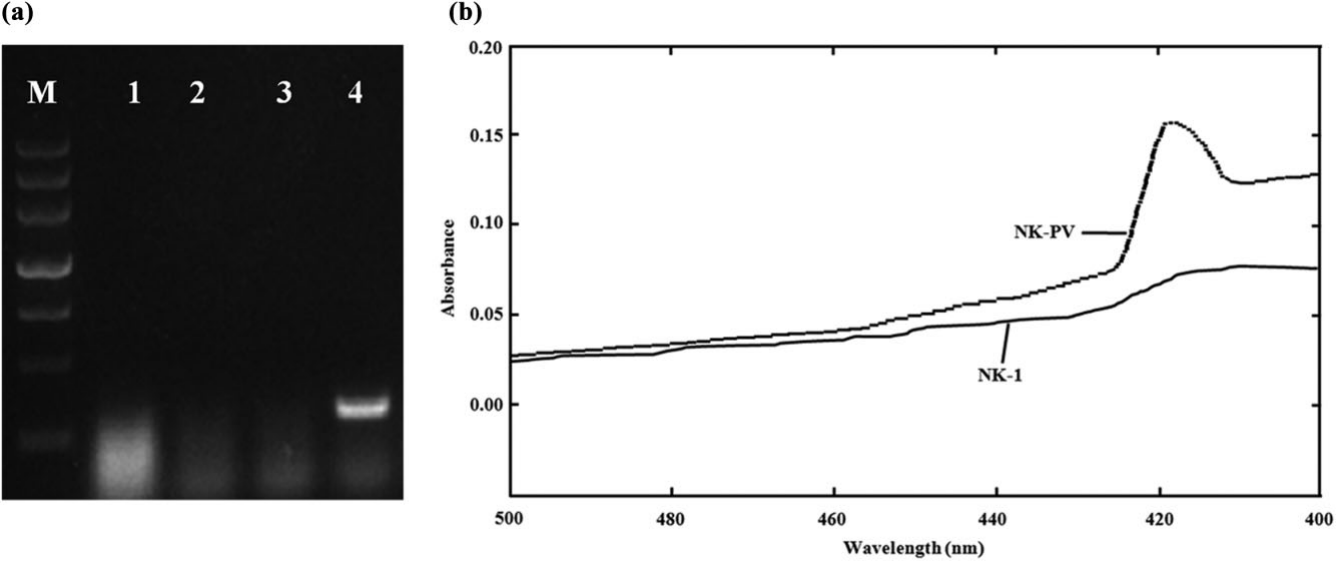
Conformation of the expression of *vgb* gene. A. RT-PCR results between NK-1 and NK-PV strain. Lane M, DNA marker III; Lane 1–4, amplification results using primers VG-F and VG-R with RNA from NK-1 strain, RNA from NK-PV strain, cDNA from NK-1 strain and cDNA from NK-PV strain as template respectively. B. CO-spectra difference analysis of NK-1 and NK-PV strain.

It has been reported that VHb expression enhances the cell density and yield of metabolite production (Olano *et al*., 2008). Here, we inserted the *vgb* gene in the *AmyA* locus of the NK-7 chromosome. To identify expression of the *vgb* gene, RT-PCR and carbon monoxide (CO)-difference spectra were used. The *vgb* gene transcriptional levels of the NK-PV strain were tested: It was transcribed successfully (Fig. 2A). VHb has been reported to bind with CO and to show a characteristic absorption peak at 420 nm (Liu and Webster, 1974). CO-difference spectra of the NK-PV strain showed an absorption peak at approximately 420 nm after 36 h of induction, but this peak was absent in the NK-1 strain (Fig. 2B). These results suggested that the mutant NK-PV strain bearing the *vgb* gene could express functional VHb.

**Figure 3:**
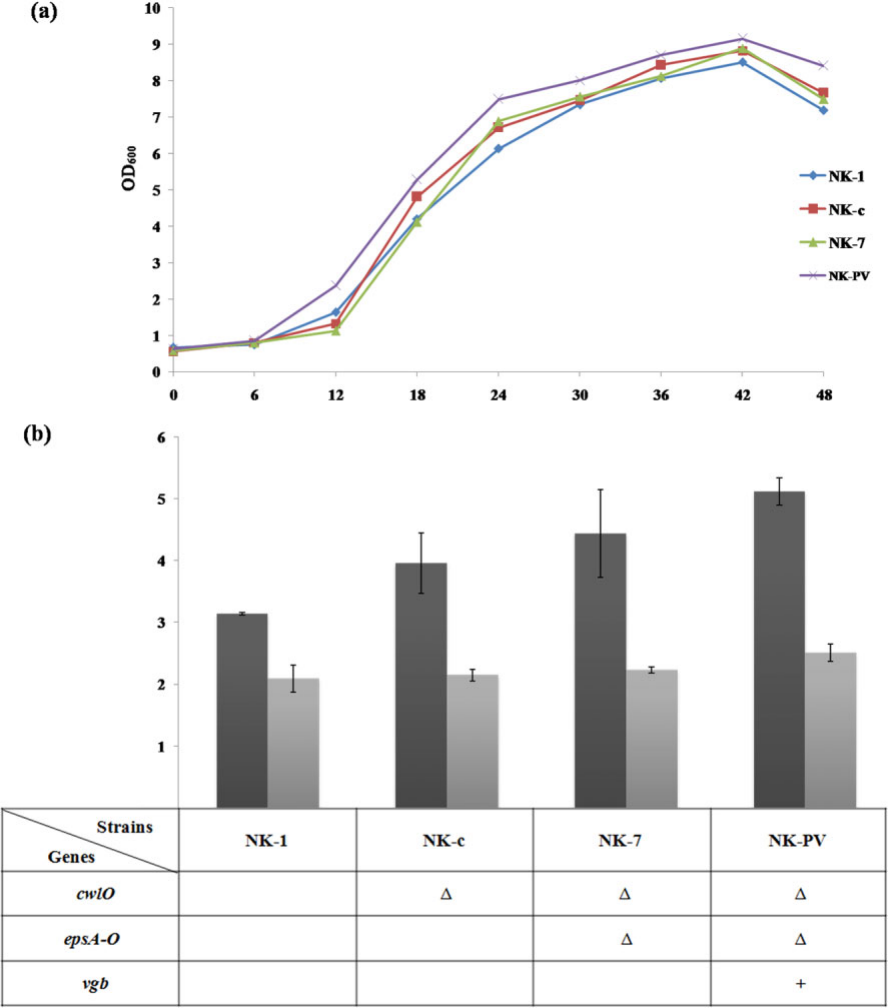
γ-PGA fermentation results between NK-1 and mutant strains. A. Time curves of cell growth of NK-1 and mutant strains. B. Comparison of γ-PGA production and cell dry weight between NK-1 and mutant strains after 48 h cultivation. Values represent means ± SD.

To confirm the effect of VHb on cell growth, the growth curve of *B. amyloliquefaciens* NK-1 and mutant strains were monitored by measuring OD_600_. The growth rate of NK-PV was higher than that of other strains without the *vgb* gene (Fig. 3A). The dry cell weight of the NK-PV strain was also higher than that of the other mutant strains. These results suggested that VHb expression increased cell growth and dry cell weight in our strain.

### Comparison of the results of γ-PGA fermentation among *B. amyloliquefaciens* NK-1 and mutant strains by flask culture

To evaluate accumulation of gene-targeted *B. amyloliquefaciens* mutants on γ-PGA production, flask culture of *B. amyloliquefaciens* NK-1 and gene mutant strains NK-c, NK-7 and NK-PV was undertaken under identical conditions. The results of γ-PGA fermentation among *B. amyloliquefaciens* NK-1 and the other mutant strains are shown in Fig. 3B and Table 1. The γ-PGA yield from NK-c was approximately 26.2% higher than that of the NK-1 strain. The molecular weight was also higher than that of the NK-1 strain. These results were in accordance with a study carried out in the glutamate-dependent strain, in which deletion of *cwlO* gene increased not only the γ-PGA yield but also its molecular weight (Mitsui *et al*., 2011). γ-PGA yields of the NK-7 strain increased by 41.5% compared with that of the NK-1 strain, and by 12.1% compared with that of the NK-c strain. The NK-PV strain showed the highest production of γ-PGA, leading to a yield of 5.12 g l^−1^, which was 63.2% higher than that of the NK-1 strain (3.14 g l^−1^). The molecular weight of γ-PGA in NK-7 and NK-PV strains was also higher than that of the NK-1 strain (Table 1).

**Table 1:** Comparison of γ-PGA fermentation results between NK-1 and mutant strains

Strains	Yield (g l^−1^)	Mw (× 10^5^)	Viscosity (cP)	CDW (g l^−1^)	Purity (%)
NK-1	3.14 ± 0.02	3.39 ± 0.08	10.05 ± 3.31	2.09 ± 0.22	76.8 ± 1.1
NK-c	3.96 ± 0.49	4.12 ± 0.04	16.70 ± 1.85	2.15 ± 0.09	75.7 ± 1.7
NK-7	4.44 ± 0.71	4.01 ± 0.14	19.40 ± 2.40	2.24 ± 0.05	81.8 ± 0.8
NK-PV	5.12 ± 0.22	3.76 ± 0.49	31.57 ± 1.33	2.51 ± 0.14	80.4 ± 1.3

The viscosity of the culture broth of the NK-1 strain and other mutant strains was 10.05, 16.70, 19.40 and 31.57 (cP) respectively (Table 1). Our previous studies have shown that a higher concentration or higher molecular weight of γ-PGA leads to a more viscous broth. In the present study, the molecular weight of γ-PGA in gene mutant strains was similar whereas the γ-PGA yield changed considerably. The strain with the higher concentration of γ-PGA made the broth more viscous. Thus, even though several factors affect broth viscosity, the viscosity of the culture broth in these fours strains was mainly determined by its γ-PGA component.

We further determined the fermentation results between NK-1, NK-c, NK-PV, NK-c-HB and NK-PV-HB strains. γ-PGA yield and molecular weight of NK-1 strain were 3.4 g l^−1^ and 391.5 K respectively. The γ-PGA yields of *cwlO* gene complementary strains NK-c-HB (3.32 g l^−1^) and NK-PV-HB (3.73 g l^−1^) are all lower than their corresponding gene deletion strains NK-c (4.35 g l^−1^) and NK-PV (5.33 g l^−1^). Moreover, molecular weights of NK-c-HB and NK-PV-HB strains are 379.7 k and 401.2 k, which were all lower than NK-c (455.6 k) and NK-PV (440.1 k).

### Effect of *epsA-O* deletion on biofilm formation

**Figure 4:**
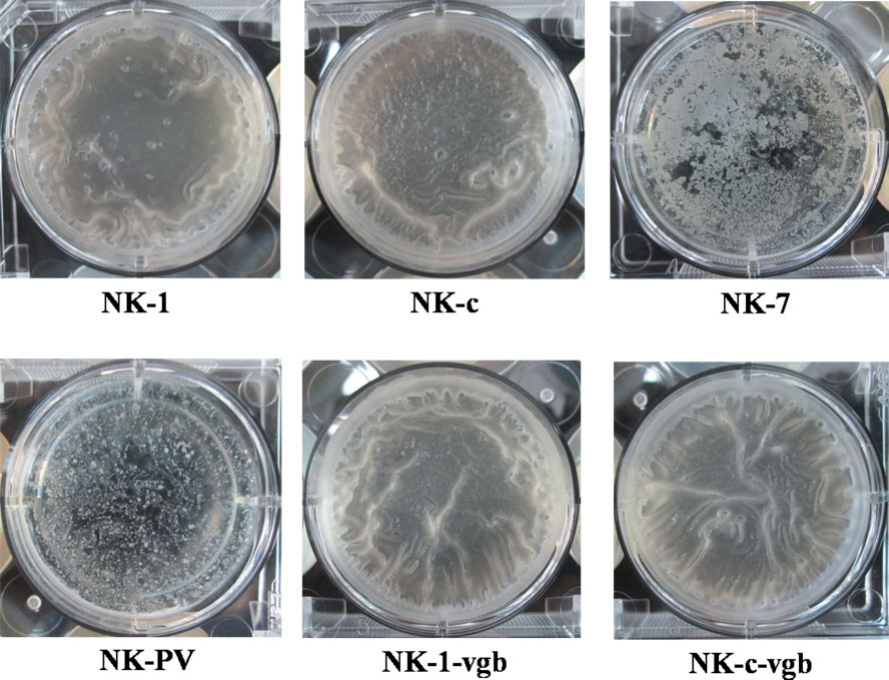
Cell pellicle formation between NK-1 and mutant strains. Cells were cultured at 30°C for 80 h in MSgg broth contained within a 6-well microtiter dish.

Microbes can construct structurally complex biofilms through production of an extracellular matrix. Studies have shown that a wild-type strain of the Gram-positive bacterium *B. subtilis* can build such a matrix. Some genetic, biochemical and cytological evidence have suggested that the matrix is composed predominantly of a protein component (TasA) and an EPS component, and that absence of TasA or the EPS results in a residual matrix, and that absence of both components leads to complete failure of biofilm formation (Branda *et al*., 2004; 2006). The *epsA-O* operon has been reported to encode biosynthetic machinery to produce EPS (Branda *et al*., 2004; Kearns *et al*., 2005). Once the *epsA-O* cluster is disrupted*, Bacillus* strains cannot secrete EPS, which greatly affects biofilm formation. In this work, the *epsA-O* cluster was deleted and its affect on biofilm formation determined. The NK-1 strain and NK-c strain could form a complete pellicle (Fig. 4). The NK-7 strain and NK-PV strain could only form a deficient incomplete pellicle.

### Effect of *epsA-O* deletion on γ-PGA purity and colony morphology

**Figure 5:**
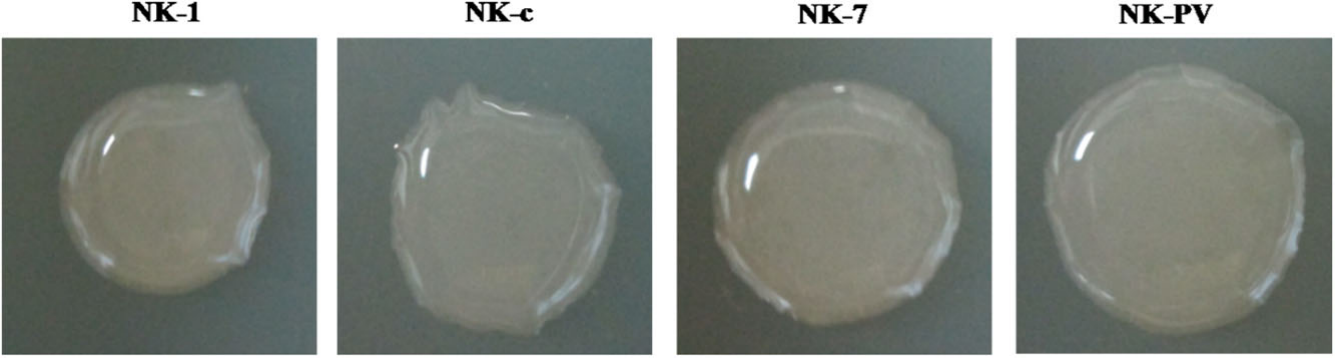
Colony morphologies of NK-1 and mutant strains. Cells were cultured at 37°C for 36 h in LB agar plate.

Some EPS can also be deposited by cold ethanol and, upon mixing with γ-PGA, decrease the purity of the γ-PGA product. To increase the purity of γ-PGA, we knocked out the *epsA-O* cluster. The purities of γ-PGA in the *epsA-O* cluster-deleted strains NK-7 and NK-PV were 81.8% and 80.4% respectively, which were higher than those of NK-1 and NK-c strains (76.8% and 75.7% respectively). The colony morphologies of NK-1 and gene mutant strains are shown in Fig. 5. The colonies of different strains were similar, only colonies of the NK-PV strain showed a slightly flatter and larger appearance than the other strains.

## Discussion

Most γ-PGA-producing *Bacillus* strains are unsuitable for genetic manipulation because of their low or absent genetic competence (Sung *et al*., 2005). Thus, few metabolic engineering strategies have been reported to improve γ-PGA production. The temperature-sensitive shuttle plasmid pKSV7 and *upp* counter selection-based marker-less deletion method has been used successfully for the manipulation of multiple genes (Smith and Youngman, 1992; Keller *et al*., 2009). In this work, we deleted the *cwlO* and *epsA-O* clusters and inserted a *vgb* gene in the NK-1 chromosome based on this method. The final metabolically engineered *B. amyloliquefaciens* NK-PV strain showed increased production of γ-PGA as well as increased purity.

Certain studies have focused on improving γ-PGA production by deletion of its degrading enzyme genes, but the *ggt* and *pgdS* gene deletion mutant strain produced similar amounts of γ-PGA to the wild-type strain (Kimura *et al*., 2004; Mitsui *et al*., 2011). This may be because GGT and PgdS are regulated appropriately *in vivo* and they function only if nitrogen in the medium is limited. Mitsui and colleagues (2011) investigated the effects of the cell wall lytic enzymes, LytE, LytF, CwlO and CwlS, on γ-PGA production in *B. subtilis* (natto). They found that disruption of the *lytE*, *lytF* and *cwlS* genes had little effect on γ-PGA production, whereas γ-PGA levels in the *cwlO* mutant were approximately twofold higher than that of the wild-type. Furthermore, they found that the *cwlO* deletion strain showed a higher molecular weight of γ-PGA. *cwlO* deletion stops γ-PGA being hydrolysed by CwlO, and its absence results in a higher yield and molecular weight of γ-PGA. Domínguez-Cuevas and colleagues (2013) reported that the *cwlO* gene deletion strain exhibits a shorter and wider cell size, resulting in a higher specific surface area that could increase the γ-PGA transport and thereafter the production of γ-PGA. In the present study, the *cwlO* gene deletion strain NK-c showed a higher yield and molecular weight of γ-PGA compared with the NK-1 strain, which was consistent with our previous work (Feng *et al*., 2014) and that of Mitsui and colleagues (2011); moreover, the *cwlO* gene complementary NK-c-HB and NK-PV-HB strains exhibited lower yield and molecular weight of γ-PGA. These results indicated that CwlO indeed related to the γ-PGA hydrolyse. No report has demonstrated that deletion of the *epsA-O* cluster or expression of the *vgb* gene increases the molecular weight of γ-PGA, so the higher molecular weights of γ-PGA in NK-c, NK-7 and NK-PV strains must be related mainly to deletion of the *cwlO* gene.

Bacteria can produce a wide variety of EPS such as curdlan (Shih *et al*., 2009), alginate (Celik *et al*., 2008), xanthan (Kalogiannis *et al*., 2003) and levan (Shih *et al*., 2010). The biosynthesis of EPS involves consumption of a large amount of the carbon source, which decreases the metabolic flux used for γ-PGA production. Moreover, EPS are secreted into the medium and interact with γ-PGA, thereby influencing the purity of γ-PGA. Liu and colleagues (2011) depressed EPS production in *B. amyloliquefaciens* C06, which caused overproduction of its γ-PGA. We deleted the *epsA-O* cluster based on the NK-c strain: The generated NK-7 strain showed a higher yield and purity of γ-PGA. Disruption of the *epsA-O* cluster blocks the pathway of EPS synthesis, which reduce the production of extracellular products as well as enables the metabolic flux to be used more efficiently for γ-PGA production. Moreover, defects in EPS production would reduce spatial competition and cell membrane permeability pressure, which would enable intracellular γ-PGA to be transported more efficiently and to accumulate outside cells. Thus, the *epsA-O* deletion strain showed good properties for γ-PGA production.

In *Bacillus* strains, EPS and TasA are two major components of the extracellular matrix that hold differentiated cell chains together to form a highly organized colony and pellicle architecture. Branda and colleagues (2006) demonstrated that single deletion of the *eps* cluster can result in incomplete formation of biofilms, and that double-deletion of *eps* and *tasA* ensure that the strain fails to make a pellicle. In the present study, the wild-type strain and *cwlO* deletion strain could form complete pellicles. In accordance with the previous study, the *epsA-O* deletion strains NK-7 and NK-PV could form only an incomplete pellicle (Branda *et al*., 2006). However, biofilm formed by NK-PV strain seems even more incomplete than NK-7 strain (Fig. 4). To test whether the expression of VHb influences bacteria biofilm formation, we determined the biofilm formation in two *vgb* gene expression strains NK-1-vgb and NK-c-vgb. Form Fig. 4, we can find that the two strains can all form complete pellicle like NK-1 and NK-c strains. These results indicated that VHb expression has little effect on bacteria bioflim formation, and it is not efficient to affect biofilm formation in the wild-type strains.

VHb has been used widely for the enhancement of cell density and target products, especially in highly viscous media, in which oxygen availability is limited (Frey and Kallio, 2003; Su *et al*., 2010). γ-PGA synthesis results in extreme viscosity of the culture medium, which seriously limit its oxygen content. p43 is a strong and well-characterized promoter in *B. subtilis* (Wang and Dio, 1984), which is assumed to express genes efficiently in other *Bacillus* genus stains. We expressed the *vgb* gene under the p43 promoter in the present study. However, γ-PGA in the NK-PV strain increased by only 15.3% compared with the NK-7 strain. This finding could be due to several reasons. VHb elicits its effects under oxygen-limited conditions (Zhang *et al*., 2007; Su *et al*., 2010). However, the oxygen-limited condition in NK-PV fermentation appeared after 12–18 h of incubation, which reduced the time in which VHb could elicit its effects. It has been reported that VHb is more likely to elicit its effects, for example, in a greater medium volume or at a lower rotation speed. However, the γ-PGA yields under those conditions are lower than those under optimal conditions (Zhang *et al*., 2007; Su *et al*., 2010). Therefore, under our cultivation conditions, the limitation of oxygen transfer may be less severe than that in other strains.

In summary, a metabolically engineered high γ-PGA productivity strain was created in a glutamate-independent γ-PGA-producing strain. We double-deleted the *cwlO* gene and *epsA-O* cluster and inserted the *vgb* gene in the NK-1 strain chromosome. The final generated NK-PV strain showed the highest γ-PGA production, which was 63.2% higher than that of the wild-type NK-1 strain; moreover, the γ-PGA purity in NK-PV strain were also higher than that of the NK-1 strain.

## Experimental procedures

### Strains, plasmids and growth conditions

All the strains and plasmids used in this work are listed in Table 2. *Escherichia coli* DH5α was used for the propagation and transformation of plasmids. Demethylation of plasmids was carried out in *E. coli* GM2163. The p43-*vgb* gene fragment was isolated from the pKSPVK plasmid (Zhang *et al*., 2013). The oligonucleotide primers used in this study are listed in Table 3.

**Table 2:** Strains and plasmids used in this study

Strains and plasmids	Relevant genotype and characteristics	Source
Strains		
*B. amyloliquefaciens* LL3	Poly-γ-glutamic acid (γ-PGA) producing strain	(Cao *et al*., 2011)
*B. amyloliquefaciens* NK-1	LL3 derivative, ΔpMC1, Δ*upp*	This lab
*B. amyloliquefaciens* NK-c	NK-1 derivative, Δ*cwlO*	This work
*B. amyloliquefaciens* NK-7	NK-1 derivative, Δ*cwlO*, Δ*epsA-O*	This work
*B. amyloliquefaciens* NK-PV	NK-1derivative, Δ*cwlO*, Δ*epsA-O*, p43-*vgb*	This work
*B. amyloliquefaciens* NK-c-HB	NK-c derivative with complementary plasmid pWH1520-cwlO	(Feng *et al*., 2014)
*B. amyloliquefaciens* NK-PV-HB	NK-PV derivative with complementary plasmid pWH1520-cwlO	This work
*B. amyloliquefaciens* NK-1-vgb	NK-1 derivative with *vgb* gene expression plasmid pWHV	This work
*B. amyloliquefaciens* NK-c-vgb	NK-c derivative with *vgb* gene expression plasmid pWHV	This work
*E. coli* DH5α	F^-^, φ80d*lac*ZΔM1, Δ(*lacZYA-argF*)U169, *deoR*, *recA*1, *endA*1, *hsdR*17(r_k_^-^, m_k_^+^), *phoA*, *supE*44, λ^-^*thi*-1, *gyrA*96, *relA*1	This lab
*E. coli* GM2163	F^-^, *ara-14 leuB6 thi-1 fhuA31 lacY1 tsx-78 galK2*	This lab
	*galT22 supE44 hisG4 rpsL 136 (Str^r^) xyl-5 mtl-1*	
	*dam13::*Tn9 (Cam^r^) *dcm-6 mcrB1 hsdR2 mcrA*	
Plasmids		
pKSV7	Shuttle vector, temperature-sensitive (ts) replication origin, Amp^r^ (gram-negative)and Cm^r^ (gram-positive)	(Smith and Youngman, 1992)
pMD19-T	T easy vector for gene cloning; Amp^r^	Takara
pKSPVK	pKSV7 derivative consists of LL3 16S rRNA gene, promoter p43, *vgb*, and Kan^r^ for selection	(Zhang *et al*., 2013)
p-upp	pKSV7-derivation with *upp* gene	This lab
p-upp-ΔAO	pKSV7-derivation with *upp* gene and deletion fragment of *epsA-O*	This work
p-upp-ΔcwlO	pKSV7-derivation with *upp* gene and the deletion of fragment *cwlO*	(Feng *et al*., 2014)
p-upp-spvx	pKSV7-derivation with *upp* gene and insertion fragment p43-*vgb*	This work
pWHV	pWH1520 derivative, *vgb* expression vector coding for VHb	(Zhang *et al*., 2013)
pWH1520-cwlO	pWH1520 derivative carrying the structural gene *cwlO*	(Feng *et al*., 2014)

**Table 3:** Primers used in this study

Primers	Sequence(5′-3′)
AO-SF	GCC*GGATCC*AGTCGGCATTTTTTACGCCGTCC
AO-SR	CAGTCTCGATCAGACGTGTCATGATAAAAATCAGTAA
AO-XF	GATTTTTATCATGACACGTCTGATCGAGACTGCAGGCA
AO-XR	CCC*GTCGAC*ACGTAAAAACCCGGTTCCTCAT
AO-SS	GAAAAATGCGCCGCCATGAATCCATAC
AO-ZZ	CGTCTGGTCATCATCAATAAAAGCCACAGG
AO-XX	CGCGACAGATAATCTTTCGTGTCACGG
cwlO-SS	GACTGACGTCATGAGCTGCTGGGTTTTT
cwlO-XX	CCAAGTTCTTTTTCACCGGGAACGCC
Amy-SF	CCCC*GTCGAC*AATGTTGCATTAAGAAGGCTGAAAACG
Amy-SR	TGCATGCACGAAGCTCTTTCGTTTTTGAATCATTTTTCT
Amy-XF	GCGGTTGAATAATGAATATGTACGGGACAAAAGGGAC
Amy-XR	CCCC*GGATCC*TTATTTTTCCAAGGCGAAAGATT
PV-F	ATTCAAAAACGAAAGAGCTTCGTGCATGCAGGCCG
PV-R	TTGTCCCGTACATATTCATTATTCAACCGCTTGAG
Amy-SS	AGGTTTTCACCCGCATATTAAGCAG
Amy-XX	AGGACAGAAAAAACAGAAACAGCACG
VG-F	TCCTGTATTGAAGGAGCATGGCGTTAC
VG-R	CGCCTGCTTGACAATGTTTGACTGC

The underlined is the restriction enzyme cleavage site.

*B. amyloliquefaciens* and *E. coli* strains were grown at 37°C in lysogeny broth (LB) medium for the routine construction and maintenance of strains. For γ-PGA production, *B. amyloliquefaciens* was cultured at 37°C at 180 r.p.m. for 48 h in γ-PGA fermentation medium (Cao *et al*., 2011). If required, antibiotics were used (100 μg ml^−1^ ampicillin, 5 μg ml^−1^ chloramphenicol). 5-fluorouracil (5-FU) was added to the medium at a final concentration of 100 μg ml^−1^.

### DNA manipulation and construction of mutant strains

The temperature-sensitive plasmid p-upp was used to construct the *epsA-O* deletion vector. The upstream and downstream fragments of *epsA-O* were amplified by PrimeSTAR HS DNA Polymerase (Takara Bio, Tokyo, Japan) using primers AO-SF/AO-SR and AO-XF/AO-XR respectively. The two regions of DNA were joined by overlap-polymerase chain reaction (PCR). The generated fragment was restricted by *Sal*I and *Bam*HI, and cloned into the p-upp vector using the same enzymes to create the deletion plasmid p-upp-ΔAO. The *vgb* gene combined with the p43 promoter was inserted into the amylase gene *AmyA* region of the chromosome. To construct the *vgb* insertion vector, the p43-*vgb* fragment was obtained from the plasmid pKSPVK (Zhang *et al*., 2013) by primers PV-F/PV-R, and the upstream and downstream regions were amplified using primers Amy-SF/Amy-SR and Amy-XF/Amy-XR respectively. These three regions of DNA fragments were joined by overlap-PCR, and the generated fragment cloned into the pMD19-T simple vector for sequencing. The generated plasmid T-SPVX was digested with *Bam*HI and *Sal*I, and the fragment cloned into the p-upp vector to generate the insertion vector p-upp-spvx.

Gene knockout mutant strains were constructed by a gene marker-less deletion method based on the *upp* cassette (Keller *et al*., 2009). The *cwlO* deletion strain NK-c has been constructed in our previous work (Feng *et al*., 2014). p-upp-ΔAO and p-upp-spvx plasmids were first introduced into *E. coli* GM2163 for demethylation. Plasmids isolated from *E. coli* GM2163 were treated with *Bam*HI methyltransferase before being transformed into target strains (Feng *et al*., 2013). Cells were incubated on LB agar plates with 5 μg ml^−1^ chloramphenicol. Single colonies were picked, and primers AO-SS/AO-ZZ and Amy-SS/Amy-XX used to determine single-cross clones by PCR. Selected single-cross clones were then incubated in LB medium, supplemented with 100 μg ml^−1^ 5-FU, for 24 h at 42°C. Cells were diluted 10^5^ times and spread on LB agar plates with 5-FU. Single colonies were picked, and primers AO-SS/AO-XX and Amy-SS/Amy-XX used to determine the gene mutant clones by PCR. Double deletion of *cwlO* and *epsA-O* strains was designated ‘*B. amyloliquefaciens* NK-7’. Double deletion of *cwlO* and *epsA-O* combined with the *vgb* gene insertion strain was designated ‘*B. amyloliquefaciens* NK-PV’.

### Production of γ-PGA by flask culture

*B. amyloliquefaciens* NK-1, *B. amyloliquefaciens* NK-c, *B. amyloliquefaciens* NK-7, *B. amyloliquefaciens* NK-PV, *B. amyloliquefaciens* NK-c-HB and *B. amyloliquefaciens* NK-PV-HB strains were first incubated in 50 ml LB medium respectively, in a 250-ml flask, and cultured aerobically for 16 h at 37°C with shaking at 180 r.p.m. One milliliter seed culture was then transferred into a 500-ml flask containing 100 ml γ-PGA fermentation medium. Flask cultures were incubated at 180 r.p.m. for 48 h at 37°C. For inducible expression, 0.5% xylose was added to the gene complementary strains medium after 3 h incubation. All cultivations were repeated at least five times.

### Reverse transcription-polymerase chain reaction (RT-PCR) analyses

RT-PCR was undertaken to test expression of the *vgb* gene between NK-1 and NK-PV strains. Cells were harvested for RNA extraction after 16 h of cultivation in LB medium. The commercial RNApure Bacteria kit (Cwbio, Beijing, China) was used to extract total RNA. cDNA was obtained by reverse transcriptional synthesis with a HiFi-MMLV cDNA kit (Cwbio). Primers VG-F and VG-R were used to determine gene transcription.

### Carbon monoxide-difference spectral analysis

VHb activity was detected by CO-difference spectra. Hemoglobin can react with CO. The complex comprising CO and reduced hemoglobin has a characteristic peak at 420 nm (Choi *et al*., 2003; Zhang *et al*., 2013). *B. amyloliquefaciens* NK-1 and *B. amyloliquefaciens* NK-PV were harvested by centrifugation at 8000 r.p.m. for 20 min at 4°C after 36-h cultivation in γ-PGA fermentation medium. Cell pellets were washed with 0.1 M potassium phosphate buffer (pH 7.2) thrice. Cells were re-suspended in 20 ml of the same buffer, and were broken with a sonicator on ice (600 W for 20 min with cycles of sonication of 30 s each and 30 s pause). The crude extract was centrifuged at 8000 r.p.m. for 20 min at 4°C to remove cell debris. Samples were divided into two aliquots. One was exposed to CO for 2 min and the other to air. Then, hemoglobin levels were obtained by CO-difference spectra using a UV-1800 spectrophotometer (Shimadzu, Kyoto, Japan).

### Analyses of formation of colonies and biofilms

Bacteria were grown on LB agar for 24 h and then incubated in LB medium to an optical density at 600 nm (OD_600_) of 1.0. For analyses of colony architecture, 2 μl of starting culture were spotted onto LB agar plates for 24 h at 37°C. For analyses of pellicle formation, 10 μl of starting culture were added to 10 ml of MSgg broth (Branda *et al*., 2006) contained within a six-well microtiter dish, and the dish incubated for 80 h at 30°C without agitation. For inducible expression of *vgb* gene, 0.5% xylose was added to MSgg medium. All colonies and pellicles were photographed using a digital camera equipped with a close-up lens (Canon, Tokyo, Japan).

### Analytical procedures

γ-PGA was purified by a method described previously (Kubota *et al*., 1993). The OD of the fermentation broth was measured by the UV-1800 spectrophotometer (Shimadzu). The molecular weight of γ-PGA was measured by a gel permeation chromatography system (Cao *et al*., 2010). Relative viscosity was measured by a DV-I Digital Rheometer (Brookfield, Middleborough, MA, USA) fitted with a spindle S00 code at the shear rate of 10 r.p.m. (25°C). Whole polysaccharide content (%) in the γ-PGA product was measured by the phenol-sulfuric acid method (DuBois *et al*., 1951). The purity of γ-PGA (%) was defined as 1-polysaccharide content (%).
